# Neuronal Oxidative Stress Promotes α-Synuclein Aggregation In Vivo

**DOI:** 10.3390/antiox11122466

**Published:** 2022-12-15

**Authors:** Seok Joon Won, Rebecca Fong, Nicholas Butler, Jennifer Sanchez, Yiguan Zhang, Candance Wong, Olive Tambou Nzoutchoum, Annie Huynh, June Pan, Raymond A. Swanson

**Affiliations:** 1 Department of Neurology, University of California at San Francisco, San Francisco, CA 94158, USA; 2 Neurology Service, San Francisco Veterans Affairs Medical Center, San Francisco, CA 94121, USA

**Keywords:** Parkinson’s disease, cysteine, α-synuclein, oxidative stress, aggregate, proximity ligation assay, motor function

## Abstract

Both genetic and environmental factors increase risk for Parkinson’s disease. Many of the known genetic factors influence α-synuclein aggregation or degradation, whereas most of the identified environmental factors produce oxidative stress. Studies using in vitro approaches have identified mechanisms by which oxidative stress can accelerate the formation of α-synuclein aggregates, but there is a paucity of evidence supporting the importance of these processes over extended time periods in brain. To assess this issue, we evaluated α-synuclein aggregates in brains of three transgenic mouse strains: hSyn mice, which overexpress human α-synuclein in neurons and spontaneously develop α-synuclein aggregates; EAAT3^−/−^ mice, which exhibit a neuron-specific impairment in cysteine uptake and resultant neuron-selective chronic oxidative stress; and double-transgenic hSyn/EAAT3^−/−^ mice. Aggregate formation was evaluated by quantitative immunohistochemistry for phosphoserine 129 α-synuclein and by an α-synuclein proximity ligation assay. Both methods showed that the double transgenic hSyn/EAAT3^−/−^ mice exhibited a significantly higher α-synuclein aggregate density than littermate hSyn mice in each brain region examined. Negligible aggregate formation was observed in the EAAT3^−/−^ mouse strain, suggesting a synergistic rather than additive interaction between the two genotypes. A similar pattern of results was observed in assessments of motor function: the pole test and rotarod test. Together, these observations indicate that chronic, low-grade neuronal oxidative stress promotes α-synuclein aggregate formation in vivo. This process may contribute to the mechanism by which environmentally induced oxidative stress contributes to α-synuclein pathology in idiopathic Parkinson’s disease.

## 1. Introduction

Several lines of evidence indicate that α-synuclein aggregates or oligomers contribute to Parkinson’s disease [1,2,3,4]. Evidence also indicates that oxidative stress contributes to the pathogenesis of Parkinson’s disease [5]. While α-synuclein aggregation and oxidative stress may act by independent mechanisms, they may also converge on a common final pathway. One potential mode of convergence involves the effects of oxidative stress on α-synuclein aggregation. Studies using in vitro preparations have identified mechanisms by which oxidative stress can accelerate the formation of toxic aggregates [6,7,8,9,10,11,12,13] but there is a paucity of evidence that chronic oxidative stress contributes to aggregate formation in vivo. In this study we address this issue using a transgenic mouse strain that exhibits chronic neuronal oxidative stress.

α-synuclein exists in neurons as monomeric species in equilibration with ordered tetramers, and in both forms the protein conformation is likely dependent upon association with lipid membranes [1,14,15]. Higher order soluble α-synuclein aggregates are cytotoxic, and likely a major factor driving cell death and disease progression in Parkinson’s disease [16,17,18,19,20]. Oxidative modifications of α-synuclein, including nitrosylation, tyrosine dimerization, methionine oxidation, and conjugation with the lipid peroxidation product 4-hydroxynonenal, have been shown to promote α-synuclein aggregation in either cell culture or cell-free conditions [6,7,8,9]. Oxidative stress may also promote α-synuclein aggregation by more indirect mechanisms. Both proteasomal and autophagic protein degradation can be impaired by oxidative stress [10,11,12,13], and oxidative DNA damage can promote α-synuclein aggregation by both activating the c-Abl protein kinase [19,20] and stimulating formation of poly(ADP-ribose) [18].

A challenge to evaluating oxidative stress effects on α-synuclein aggregation in vivo is the dearth of ways to generate chronic oxidative stress in brain, and particularly chronic oxidative stress selectively in neurons. One way this can be accomplished is with mice deficient in excitatory amino acid transporter-3 (EAAT3; also termed EAAC1 and SLCa17). EAAT3 is the major route by which neurons uptake cysteine, which is the rate-limiting step for glutathione synthesis in mature neurons (Aoyama et al., 2006) but not other cell types. Mice deficient in EAAT3 have reduced neuronal glutathione levels, increased basal levels of neuronal oxidative stress, and increased neuronal susceptibility to exogenous oxidants [21,22,23,24]. EAAT3^−/−^ mice thus provides a useful model for evaluating cross-talk between chronic neuronal oxidative stress and α-synuclein aggregation.

The Line 61 Thy-1 α-synuclein mouse strain developed by E. Masliah and colleagues [25] over-expresses human α-synuclein under control of the neuronal Thy-1 promoter. This strain spontaneously develops neuronal α-synuclein aggregates in several brain regions and also develops motor and behavioral abnormalities [26]. Here, we investigated whether mice of this strain (henceforth termed the “hSyn” strain for brevity) exhibit an increase in aggregate formation or motor deficits when crossed with EAAT3^−/−^ mice. Our results show that both α-synuclein aggregate formation and motor impairment are exacerbated in hSyn mice lacking EAAT3, thus supporting the contention that α-synuclein aggregation in vivo is accelerated by concomitant oxidative stress.

## 2. Materials and Methods

### 2.1. Mouse Breeding and Husbandry

Line 61 Thy-1 α-synuclein (hSyn) mice on the DBA/2 strain were obtained from Dr. Elezier Masliah, UCSD. The hSyn mice carry the human α-synuclein transgene on the X chromosome (X^hSyn^). We initially backcrossed these mice onto the C57BL/6 strain (Jackson Labs), but due to poor breeding on that background the mice were re-crossed with the DBA/2 parental strain to generate a colony on a 1:1 DBA/2: C57BL/6 background, as described previously [26,27,28]. EAAT3^−/−^ mice on the C57BL/6 background were then likewise crossed with DBA/2 mice to generate a colony on a 1:1 DBA/2: C57BL/6 background. The hSyn and EAAT3^−/−^ mice were then interbred with repeated intercrosses for 6 generations to generate a colony with a homogeneous 1:1 DBA/2: C57BL/6 background. Female XX^hSyn^ mice have a very attenuated phenotype [26], and so only male mice were used for experiments. Mice used for experiments were generated by crossing EAAT3^-/+^, XY males with EAAT3^-/+^, X^hSyn^X females to produce male mice with the four genotypes of interest; wild type (EAAT3^+/+^, XY); hSyn (EAAT3^+/+^, X^hSyn^Y); EAAT3^−/−^ (EAAT3^−/−^, XY); and hSyn/EAAT3^−/−^ (EAAT3^−/−^, X^hSyn^Y). The numbers of each genotype used for the experiments are listed on Table 1, along with weight and mortality data. Mice of the hSyn/EAAT3^−/−^ genotype exhibited greater mortality over the 8 month experimental period than the other mouse genotypes (Table 1).

### 2.2. Experimental Design

Studies were approved by the San Francisco Veterans Affairs Medical Center animal studies committee and performed in adherence to the Guide to the Care and Use of Experimental Animals and The Ethics of Animal Experimentation and the 2020 ARRIVE guidelines [29]. A total of 71 mice were assessed, divided into 4 cohorts of 16–20 mice, each with a roughly equal distribution of genotypes. Each cohort of mice underwent behavioral assessments first at age 3–4 months and then again at age 7–8 months. At age 8 months the mice were euthanized and brains removed for histology. All brains from each cohort were fixed, immunostained, and analyzed in a single batch.

### 2.3. Behavioral Assessments

Each cohort of mice was assessed using the rotarod test and the pole test as described [19,30], with minor modifications. The individuals handling and scoring the mice were blinded to mouse genotype. For habituation to the rotarod test, mice were placed for 90 s on a horizontal rod (San Diego Instruments Rota-rod) rotating at 4 rpm (1st day) and 5.5 rpm (2nd day), 5 times each day. For testing, mice were placed on the horizontal rod at a start speed 0 rpm and an acceleration rate of 6 rpm/minute, and the time until fall from the rod was recorded. Tests were repeated 6 times each day for 2 consecutive days. The lowest and highest time for each mouse was discarded and the average time from the remaining 10 trials was calculated for each mouse. For the pole test, mice were placed face-up at the top of a pole (45 cm length, 1.2 cm diameter), wrapped in tape for grip, and scored for turn-around time (time taken to face downward) and descent time (time taken to come down the pole). A cardboard square was affixed to the top of the pole to prevent mice from crossing up and over the pole to descend. Each assessment involved 2 days of habituation followed by 3 days of testing. Testing was performed twice per day every other day and video recorded. The recordings were later analyzed by an observer who was also blinded to the treatment conditions. Timing was begun with the first mouse movement, and any time exceeding 3 s during which the mouse was motionless was subtracted from the total time. The longest and shortest times were discarded, and the average time from the remaining 10 trials was calculated for each mouse. Descent-time was not used for data analysis because some impaired mice were unable to descend the pole normally and instead either slid down or used their tails to assist. Results obtained from mice that died before the second behavioral assessment were also excluded from the data analysis.

### 2.4. Immunohistochemistry and Image Analysis

Deeply anesthetized mice were perfused with cold 0.9% NaCl followed by perfusion with freshly depolymerized paraformaldehyde (PFA; 4%) in 0.1 M sodium phosphate buffer (PB) pH 7.0. The brains were removed and post-fixed in the same PFA/PB solution for 18 h, followed by incubation for 24 h in 20% sucrose for cryoprotection. The brains were then frozen and coronal sections (40 µm thickness) were prepared with a cryostat. For immunohistochemistry, the sections were incubated in blocking buffer (2% donkey serum and 0.1% bovine serum albumin in 0.1 M PB) at room temperature for 30 min, and then incubated with the primary antibodies overnight at 4 °C. The antibody sources and dilutions used are listed in Table 2. After washing, antibody binding was detected using fluorescent secondary antibodies. The sections were then mounted on glass slides in DAPI-containing mounting medium (Vector laboratories, Burlingame, CA). Controls were prepared omitting either primary or secondary antibodies.

Immunostaining for phosphoserine-129 α-synuclein (pSyn) was quantified in cortex and hippocampus in evenly spaced sections spanning bregma −1.5 to −2.3. The sections were immunostained with anti-pSyn and anti-NeuN. Confocal images were taken from both the right and left hemisphere of a pre-determined region of cortex (layer 5) and hippocampus CA1 on each of three sections from each animal. Two optical sections through each region were stacked in Image J software. Neuronal perikarya as identified by NeuN staining were used as a mask to identify the regions of interest, and the mean intensity of anti-pSyn immunofluorescence within these regions was measured. For analysis of the substantia nigra, sections were additionally immunostained for tyrosine hydroxylase (TH) to permit assessment specifically in dopaminergic neurons. The substantia nigra pars compacta was defined by the pattern of the TH-positive neurons, as previously described [19,31]. Confocal images were taken at both right and left hemisphere of substantia nigra from three sections spaced 240 µm apart (bregma −2.8 to −3.6). Two optical sections through each region were stacked in Image J software. The TH-positive neurons were identified in the confocal images and NeuN-demarcated perikarya of the TH-positive cells was used as a mask to identify the regions of interest. The measurements made in the left and right cortex of all three sections were then averaged to generate a single value for the cortex of each brain, and the same was done for hippocampus and substantia nigra. To minimize potential differences introduced by variations in fixation, antibody binding, or photography, all values from each cohort were normalized to the mean of the wild-type values measured in the cohort. Both the persons taking the photographs and analyzing the images were blinded to the mouse brain genotypes.

### 2.5. Proximity Ligation Assay

In two cohorts of mice, α-synuclein density was additionally analyzed using a proximity ligation assay (PLA), as described [32]. The experiments were performed using Duolink PLA Brightfield kits from Millipore Sigma according to the manufacturer’s manual. In brief, brain sections mounted on glass slides were incubated with 10 mM sodium citrate, pH 6.0 for 10 min at 95 °C for antigen retrieval, then incubated in hydrogen peroxide to quench endogenous peroxidase activity. Antibody-oligonucleotide conjugates were prepared using the Duolink Probemaker kit by incubating mouse anti-α-synuclein with plus- or minus- sense oligonucleotide probes. The brain sections were then immersed sequentially in Duolonk ligase, polymerase and amplification reagents, and Duolink detection solution. The sections were counterstained to identify cell nuclei and dehydrated in graded ethanol and citrisolve.

In a separate study, the PLA method was used to identify α-synuclein—4-hydroxynonenal conjugates. For these studies the sections were incubated with mouse antibody to α-synuclein and rabbit antibody to 4-hydroxynonenal, as previously described [33]. After washing, the sections were incubated with anti-rabbit plus sense and anti-mouse minus sense oligonucleotide probes. The brain sections were then immersed sequentially in Duolink blocking solution, the antibody-oligonucleotide conjugates, Duolonk ligase, polymerase and amplification reagents, and Duolink detection solution.

PLA signals were quantified using a stereological microscope (Stereo Investigator, MicroBrightField, Williston, VT). Every sixth coronal section spanning the substantia nigra (Bregma level –2.5 to –3.5) was used, which includes cortex and hippocampal regions. A counting frame (25 × 25 × 20 µm) was placed at each intersection of a 200 × 200 µm sampling grid superimposed onto regions of interest manually outlined on each section. For cortex, this was a 1 mm^2^ area polygon on the dorsal surface (including layers 2–6); for hippocampus, this was a 1 mm^2^ polygon encompassing dorsal CA1, stratum oriens and stratum radiatum; and for substantia nigra the polygon was drawn using morphological landmarks [19,31]. PLA puncta in the counting frames were manually counted using 40x objective (Axioskop 2 plus; Carl Zeiss Microscopy, LLC, White Plains, NY, USA). In many instances the PLA puncta were overlapping. For this reason, the analysis was performed two ways; with only the discrete single puncta counted and then with only the overlapping clusters of puncta counted. The tissue preparation and puncta counts were performed by observers blinded to the mouse genotype. Data analysis was performed separately for single puncta and multi puncta counts, using the same method as for the pSyn immunostaining.

### 2.6. Western Blots

Brain wedges containing cortex and hippocampus were homogenized in RIPA buffer (Thermo Scientific, Rockford, IL, USA) containing Halt protease and phosphatase inhibitor cocktail (Thermo Scientific). The tissue lysate was centrifuged at 12,000× *g* for 10 min at 4 °C and 30 µg of protein was subjected to electrophoresis on NuPAGE 4–12% Bis-Tris gel (Invitrogen, Carlsbad, CA, USA) and transferred to a PVDF membrane. The blot was incubated in 10% BSA in Tris-Buffered Saline with Tween 20 (TBST) for 1 h and then incubated overnight with a primary antibody (Table 2). The blot was washed with TBST and reacted with a horseradish peroxidase secondary antibody for 1 h. The immunoreactivity was visualized by a chemiluminescent substrate (Thermo Scientific) and the signals were acquired by using a C300 imager (Azure Biosystems, Dublin, CA, USA).

### 2.7. Statistics

All data are expressed as means ± s.e.m., with the “*n*” of each study defined as the number of mice. Data were analyzed using ANOVA and Sidak’s test for multiple comparisons, or by t-tests where only two groups were compared. Normality of each data set was assessed by the Shapiro–Wilk test, and all data sets passed (did not significantly deviate from normality) at the α = 0.05 level.

## 3. Results

**Figure 1:**
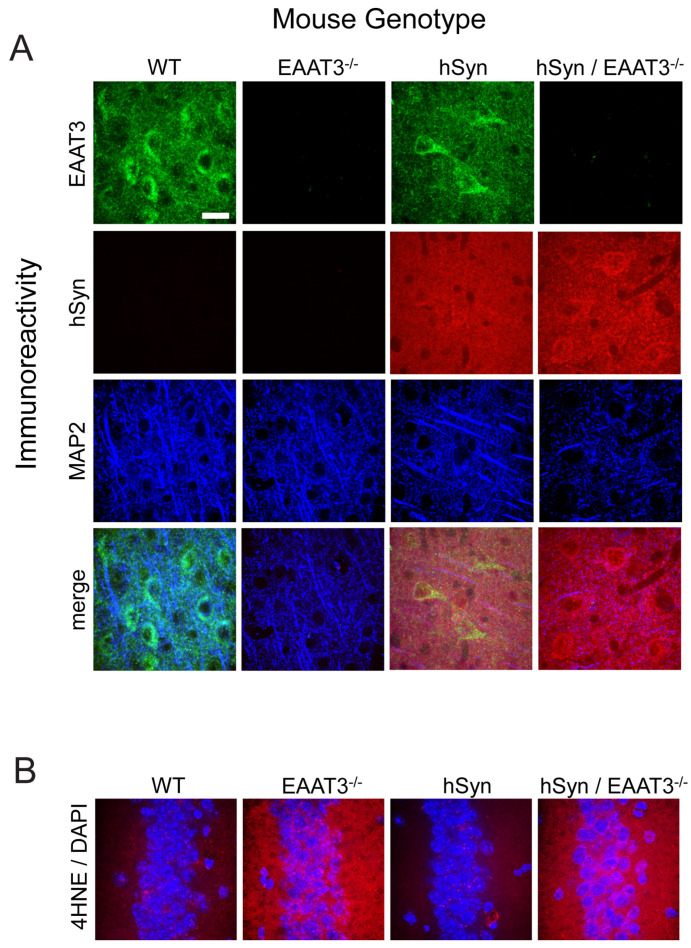
Expression of EAAT3 and human α-synuclein in the wild-type and transgenic mouse strains. (**A**) Mouse cerebral cortex immunostained for excitatory amino acid transporter-3 (EAAT3, green), human α-synuclein (hSyn, red), and the neuronal marker microtubule-associated protein-2 (MAP2; blue). The mouse strains are wild-type (WT); EAAT3^−/−^; line 61 Thy-1-hSyn (hSyn); and hSyn/EAAT3^−/−^. (**B**) Immunostaining for 4-hydroxynonenal (4HNE; red) in the hippocampal CA1 neuronal layer shows increased signal in the EAAT3^−/−^ and hSyn/EAAT3^−/−^ mice. Nuclei are stained blue with DAPI. All images are representative of *n* = 4 mice. Scale bar = 20 μm.

The four genotypes of mice generated by the transgenic crosses were not grossly distinguishable from one another and did not significantly differ in size over the age range used for these studies (Table 1). Immunostaining and Western blotting for the glutamate/cysteine transporter EAAT3 confirmed expression to be absent in the EAAT3^−/−^ and hSyn/EAAT3^−/−^ genotypes, whereas immunostaining and Western blots for human α-synuclein confirmed it to be strongly expressed in the hSyn and hSyn/EAAT3^−/−^ genotypes (Figure 1A and Figure S1). Oxidative stress was confirmed in both the EAAT3^−/−^ and the hSyn/EAAT3^−/−^ mouse strains by immunostaining for the lipid peroxidation marker 4-hydroxynonenal (Figure 1B).

**Figure 2:**
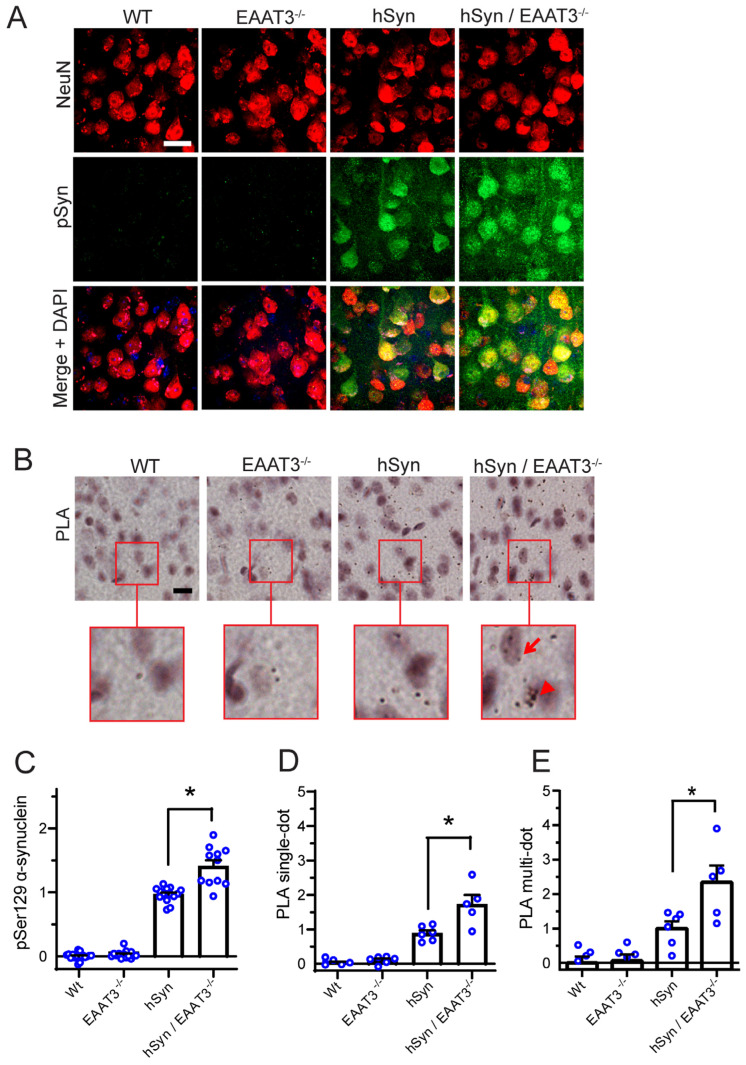
Increased α-synuclein aggregation in cortical neurons of hSyn/EAAT3^−/−^ mice. (**A**) Immunostaining for phosphoserine-129 α-synuclein (pSyn; green), with neuronal nuclei identified by NeuN (red). Nuclei of all cells are counterstained with DAPI (blue) in the merged images. (**B**) α-synuclein aggregates are identified by proximity ligation assay (PLA). Red squares demark areas shown in magnified view. Examples of single dots are shown with arrows, and multiple dots are shown with arrowheads. All scale bars = 20 μm. (**C**) Quantification of pSyn immunostaining. * *p* < 0.05, *n* = 8–11. (**D**) Quantification of single-puncta PLA signals; * *p* < 0.05, *n* = 5–7. (**E**) Quantification of multi-puncta PLA signals. For all quantifications, data from each mouse are expressed relative to signal measured in hSyn mice sections prepared and analyzed in parallel.

α-synuclein aggregates were assessed by quantitative immunohistochemistry using an antibody that recognizes both mouse and human phosphoserine-129 α-synuclein [34]. In cortical neurons, the immunostaining for phosphoserine-129 α-synuclein (abbreviated as pSer129 α-syn) revealed no detectable signal in the WT or EAAT3^−/−^ strains, but showed a robust signal in the hSyn strain as previously reported [26,27]. Notably, the pSer129 α-syn signal was increased in the hSyn/EAAT3^−/−^ mice relative to that observed in the hSyn mice (Figure 2A,C). As a confirmatory approach, aggregate formation was also assessed using a proximity ligation assay with antibodies to human α-synuclein. Aggregates are visualized as small puncta by this approach (Figure 2B, inserts). In some cases the puncta occurred in clusters, making it difficult to count the individual dots. For this reason, the data were quantified in two ways: with only discrete puncta counted, and with only clusters of puncta counted. As shown in Figure 2, the two quantification methods provided similar results and showed the same pattern as observed with the pSer129 α-syn immunostaining: a negligible signal in WT or EAAT3^−/−^ mouse cortical neurons, a robust signal in the hSyn neurons, and a significantly greater signal in the hSyn/EAAT3^−/−^ neurons (Figure 2B,D,E). A modification of the PLA method was used to determine if α-synuclein undergoes increased oxidative modification in the hSyn/EAAT3^−/−^ neurons. In this method, 4-hydroxynonenal-α-synuclein conjugates were detected using an antibodies to these two epitopes [33]. The studies revealed a greater 4-hydroxynonenal-α-synuclein PLA signal in hSyn/EAAT3^−/−^ than in hSyn brain sections (Figure S2).

**Figure 3:**
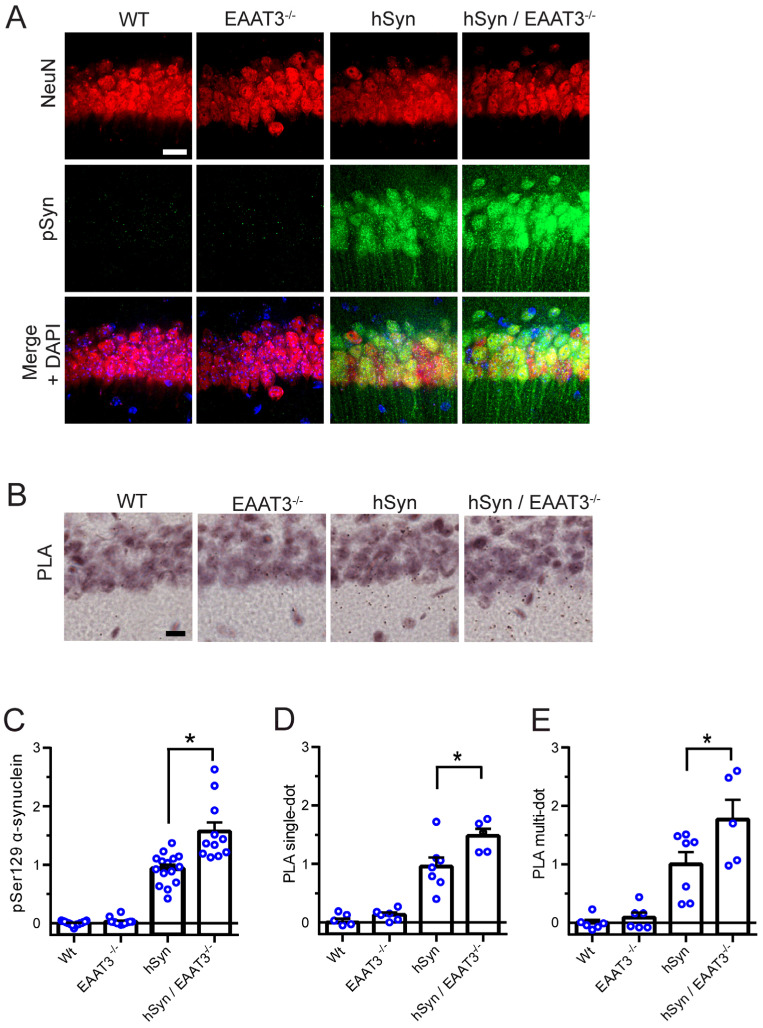
Increased α-synuclein aggregation in hippocampal CA1 neurons of hSyn/EAAT3^−/−^ mice. (**A**) Immunostaining for phosphoserine syn-129 α-synuclein (pSyn; green), with neuronal nuclei identified by NeuN (red). Nuclei of all cells are counterstained with DAPI in the merged image. (**B**) α-synuclein aggregates identified by proximity ligation assay (PLA). Scale bars = 20 μm. (**C**) Quantification of pSyn immunostaining; * *p* < 0.05, *n* = 8–11. (**D**) Quantification of single-puncta PLA signals; * *p* < 0.05, *n* = 5–7. (**E**) Quantification of multi-puncta PLA signals. For all quantifications, data from each mouse isare expressed relative to signal measured in hSyn mice sections prepared and analyzed in parallel.

hSyn mice also develop α-synuclein aggregates in neurons of the hippocampus [26], and we assessed aggregate formation in the hippocampal CA1 neurons using the same methods as for cortical neurons. We again found a good correspondence between results obtained by the two methods, and the same pattern of results was observed in the cortical neurons: a negligible signal in the WT and EAAT3^−/−^ mice, a robust signal in the hSyn mice, and a significantly greater signal in the hSyn/EAAT3^−/−^ mice (Figure 3).

**Figure 4:**
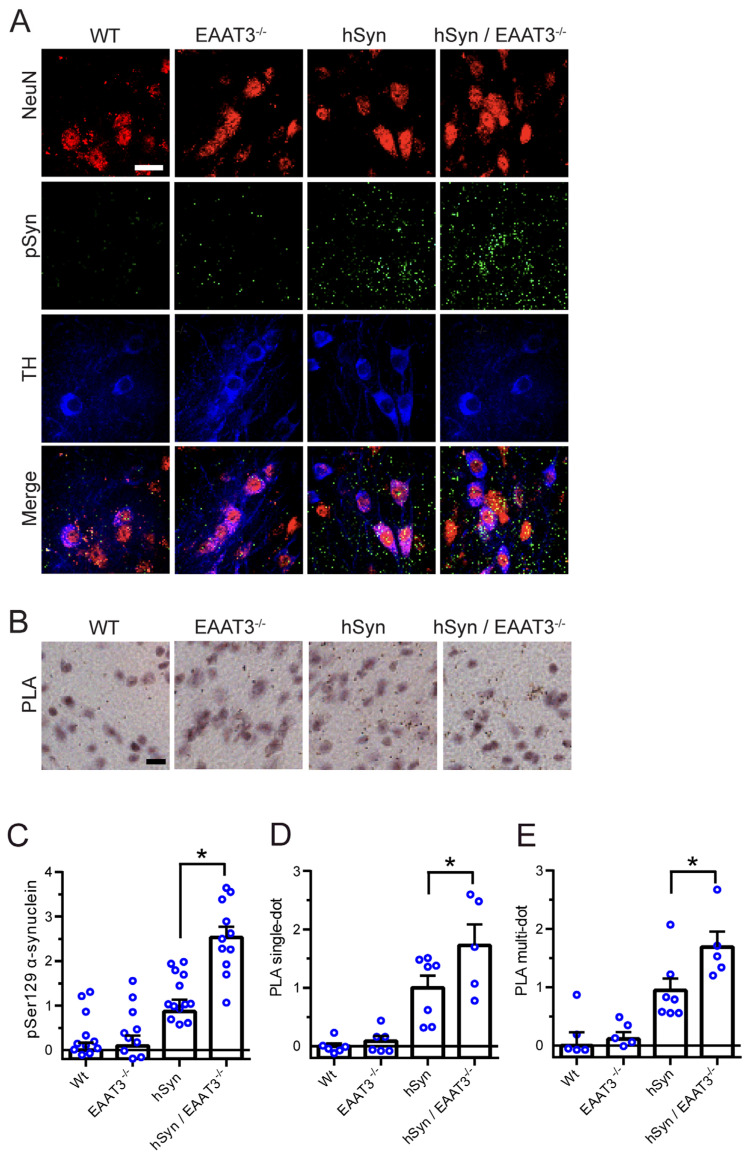
Increased α-synuclein aggregation in substantia nigra neurons of hSyn/EAAT3^−/−^ mice. (**A**) Immunostaining for phosphoserine-129 α-synuclein (pSyn; green) in dopaminergic neurons, with neuronal nuclei identified by NeuN (red) and dopaminergic neurons identified by tyrosine hydroxylase (TH; blue). (**B**) α-synuclein aggregates are identified by proximity ligation assay (PLA). Scale bars = 20 μm. (**C**) Quantification of pSyn immunostaining; * *p* < 0.05, *n* = 8–11. (**D**) Quantification of single-puncta PLA signals; * *p* < 0.05, *n* = 5–7. (**E**) Quantification of multi-puncta PLA signals. For all quantifications, data from each mouse isare expressed relative to signal measured in hSyn mice sections prepared and analyzed in parallel.

Prior reports using this hSyn mouse strain have noted less aggregate formation in the substantia nigra pars-compacta than in hippocampus or cortex, as assessed using pSer129 α-syn immunoreactivity [26,27]. Our results arere similar in that the pSyn129 signal was less robust in dopaminergic neurons of the substantia nigra pars compacta than in cortex or hippocampus (Figure 4). Nevertheless, the pattern of aggregates formation across the four mouse genotypes was the same as observed in the other structures: the pSyn129 signal was negligible in the WT or EAAT3^−/−^ mice, present in the hSyn/EAAT3^−/−^ mice, and significantly greater in the hSyn/EAAT3^−/−^ mice (Figure 4A,C). Aggregate formation assessed by the PLA assay in the substantia nigra showed the same pattern of results (Figure 4B,D,E). Of note, the magnitude of PLA signal in the substantia nigra was comparable to that observed in the cortex and hippocampus, unlike the pSyn 129 signal which was markedly less prominent in the substantia nigra than in those structures.

**Figure 5:**
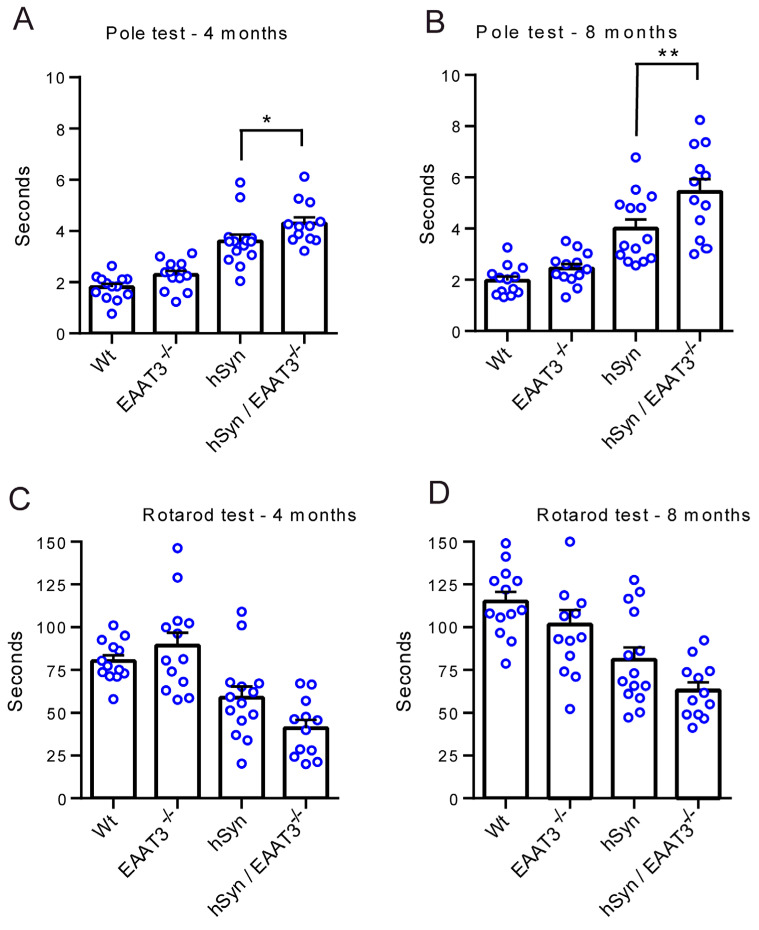
Accelerated motor decline in hSyn/EAAT3^−/−^ mice.Motor performance. (**A**,**B**) Time required for turnaround on the pole test at 4 and 8 months of age. * *p* < 0.05, ** *p* < 0.01; *n* = 8–11. (**C**,**D**) Time beforeto fall from the rotarod at 4 and 8 months of age.

These histological assessments were accompanied by two measures of motor function, the pole test and the rotarod test. The pole test revealed a small but significantly greater impairment in the hSyn/EAAT3^−/−^ mice relative to the hSyn mice at age 4 months, and a somewhat larger impairment at age 8 months (Figure 5A). Results of the rotarod test similarly showed a trend toward greater impairment in hSyn/EAAT3^−/−^ mice, though these differences did not achieve statistical significance at the *p* < 0.05 level (Figure 5B).

## 4. Discussion

These studies compared α-synuclein aggregate formation in hSyn mice, which overexpress human α-synuclein, to hSyn mice crossed with EAAT3^−/−^ mice, which in addition exhibit chronic neuron-specific oxidative stress. Both quantitative pSyn129 immunohistochemistry and a proximity ligation assay showed that the double transgenic hSyn/EAAT3^−/−^ mice exhibited significantly higher α-synuclein aggregate densities than littermate hSyn mice in each brain region examined. Negligible aggregate formation was observed in the parental EAAT3^−/−^ mouse strain, suggesting a synergistic rather than additive interaction between the two genotypes. A similar pattern of results was observed in assessments of motor function.

Many of the known genetic variants imparting risk for Parkinson’s disease affect either α-synuclein per se or aspects of protein quality control [35], while almost all environmental factors known to contribute to Parkinson’s disease generate intracellular oxidative stress [36,37]. A link between α-synuclein aggregation and oxidative stress has been established by the observations that α-synuclein aggregates themselves can induce oxidative stress [19,38], and that mice deficient in α-synuclein exhibit less dopaminergic neuronal loss when exposed to the oxidant-producing compounds MPTP or rotenone [39,40]. Moreover, cell culture studies have identified several mechanisms by which oxidative stress can accelerate α-synuclein aggregation [6,7,8,9], including conjugation of α-synuclein with the lipid peroxidation product 4-hydroxynonenal [41,42,43]. In line with those findings, the PLA assessment of 4-hydroxynonenal-α-synuclein conjugates performed here showed increased signal in the hSyn/EAAT3^−/−^ mice. However, the interpretation of this PLA finding is complicated by the fact that the hSyn/EAAT3^−/−^ neurons also contain an increased density of α-synuclein aggregates, and this increased aggregate density may contribute to the overall increase in the 4-hydroxynonenal-α-synuclein conjugate signal.

Despite the several possible mechanisms by which oxidative stress may promote α-synuclein aggregation, there have been few prior demonstrations of chronic oxidative stress influencing α-synuclein aggregation in vivo. Pharmacological studies using rotenone or MPTP (1-methyl-4-phenyl-1,2,3,6-tetrahydropyridine) to induce oxidative stress and substantia nigra dopaminergic cell loss show generally sparse evidence of α-synuclein aggregate formation [44,45,46,47,48], possibly because of the relatively short duration of these studies. A genetic approach using SOD2^+/-^ mice crossed with mice expressing the human A30P mutant α-synuclein observed an increased in α-synuclein aggregation relative to the parental A30P α-synuclein strain, as assessed by poteinase-K resistant inclusions [49]. This study differed from the present work in that the mice expressed a mutant rather than wild-type α-synuclein, and the method of aggregate detection was different. Given that SOD2 haploinsufficiency causes oxidative stress in all cell types, it is also possible that systemic or specifically microglial effects of the oxidative stress [50] rather than a direct intraneuronal mechanism may contribute to the findings in these mice. Despite these differences, the study using SOD2^+/-^ mice nevertheless comports with the present results indicating that chronic oxidative stress promotes α-synuclein aggregate formation in the intact brain.

There is evidence that EAAT3, in addition to serving as a cysteine transporter, may also function to take up glutamate at glutamatergic synapses [51]. To the extent this occurs, neurons in EAAT3^−/−^ mice could also undergo increased oxidative stress as a consequence of increased activation of NMDA-type glutamate receptors [52,53]. Additionally, elevated extracellular glutamate levels could deplete intracellular cysteine levels (and hence glutathione levels) via glutamate cystine exchange [54]. These possibilities do not significantly alter the interpretation of our results, however, because both of these potential effects of EAAT3^−/−^ would also lead to increased neuronal oxidative stress.

The patterns of α-synuclein aggregation we observed in the hSyn (Line 61) mice corresponds with prior studies using this mouse strain. As in prior reports [26,27], we found aggregate accumulation as detected by pSyn129 immunostaining to be less robust in the substantia nigra pars reticulata than in cortex or hippocampus. However, by the PLA method we found aggregates to be comparably dense in the three brain regions, consistent with aggregate assessments using other methods [55,56]. Human α-synuclein is expressed to similar levels in all three regions in hSyn mice [26]. The reason for the reduced amount of pSyn129 immunostaining signal in the substantia nigra is thus unclear, but may be attributable to cell-type differences in α-synuclein phosphorylation.

Results of our behavioral assessments also correspond to prior studies using the hSyn mice [28,57]. On the pole test, turnaround time was greater in the hSyn/EAAT3^−/−^ than in the hSyn mice at both the four-month and eight-month time points, and this difference was further increased at the eight-month time point. The double transgenic mice also did most poorly on the rotarod test, at both time points assessed, though this difference did not reach statistical significance at the *p* < 0.05 level. It is possible that the increased mortality in the hSyn/EAAT3^−/−^ mice led to an underestimate of the behavioral impairment in that genotype. Unexpectedly, all groups did slightly better at eight months than at four months on this test. The reason for this is not clear, though possibly this was a learning effect with repeated testing over time.

A limitation of our study is that accelerated neuronal death does not occur in the hSyn mice, in the substantia nigra or elsewhere, thus obviating use of that experimental endpoint. An additional limitation is that only male mice could be used, because the female mice have an attenuated phenotype [26]. However, there are only small sex differences in the incidence and progression of human Parkinson’s disease [58], and the absence of female mice in the current study should not negate the central finding that aggregate formation is promoted by chronic oxidative stress.

## 5. Conclusions

The findings of this in vivo study extend prior observations obtained using cell culture and cell-free systems, and support the contention that chronic, low-grade oxidative stress promotes the formation of α-synuclein aggregates in brain neurons. This process may contribute to the mechanism by which environmentally induced oxidative stress contributes to the α-synuclein pathology in idiopathic Parkinson’s disease.

## Figures and Tables

**Table 1:** Mouse genotype distribution and mortality.

Mouse Genotype	Mean Weight	Number Allocated	Mortality
Wild-type (EAAT3^+/+^, XY)	28.3 ± 0.8	18	1
hSyn (EAAT3^+/+^, X^hSyn^Y)	26.8 ± 0.9	20	1
EAAT3^−/−^ (EAAT3^−/−^, XY)	26.7 ± 0.7	17	2
hSyn/EAAT3^−/−^ (EAAT3^−/−^, X^hSyn^Y)	22.5 ± 0.8	16	4

Weights are means ± s.e.m. from 6 mice of each genotype at age 4 months. Mortality is the number of mice that died before the 8-month behavioral assessment.

**Table 2:** Primary antibody sources and dilutions used.

Antibody	Source	ID	Host	Dilution
NeuN	Millipore, Temecula, CA, USA	MAB377	Mouse	1:1000
pSyn (S129)	Abcam, Cambridge, MA, USA	Ab51253	Rabbit	1:1000
TH	Abcam, Cambridge, MA, USA	Ab76442	Chicken	1:1000
Synuclein (syn211)	Abcam, Cambridge, MA, USA	Ab80627	Mouse	1:750
EAAT3	Cell Signaling, Boston, MA, USA	14501s	Rabbit	1:1000
4HNE Actin	Alpha Diagnostic, San Antonio, TX, USA Sigma-Aldrich, St. Louis, MO, USA	HNE11-S A2066	Rabbit Rabbit	1:1000 1:100
anti-mouse IgG 594	Thermo Fisher Scientific, Waltham, MA, USA	A21203	Donkey	1:1000
anti-rabbit IgG 488	Thermo Fisher Scientific, Waltham, MA, USA	A21206	Donkey	1:1000
anti-chicken IgG 405	Thermo Fisher Scientific, Waltham, MA, USA	A175675	Goat	1:1000
anti-rabbit IgG, HRP-linked F(ab’)_2_	Amersham Biosciences, UK	NA934v	Donkey	1:5000
anti-mouse IgG, HRP-linked F(ab’)_2_	Amersham Biosciences, UK	NA931v	Sheep	1:5000

## Data Availability

The raw data and images that were analyzed for this publication will be provided upon request to the corresponding author: raymond.swanson@ucsf.edu

## Supplementary Materials

The following supporting information can be downloaded at: https://www.mdpi.com/article/10.3390/antiox11122466/s1, Figure S1: Western blots of EAA3 and human α-synuclein from brains of the transgenic mouse strains, Figure S2: Proximity ligation assay assessments of 4-hydroxynonenal- α-synuclein conjugates in neurons of cortex and hippocampus CA1 cell field
