# Supplementary material for: Neuronal Oxidative Stress Promotes α-Synuclein Aggregation In Vivo
Source: Antioxidants (Basel). 2022 Dec 15;11(12):2466. doi: 10.3390/antiox11122466 (PMC9774295; doi:10.3390/antiox11122466)
Supplement: Supplementary file 1 [file antioxidants-11-02466-s001.zip › antioxidants-2055697-supplementary.pdf]

**Supplemental Figure S1**

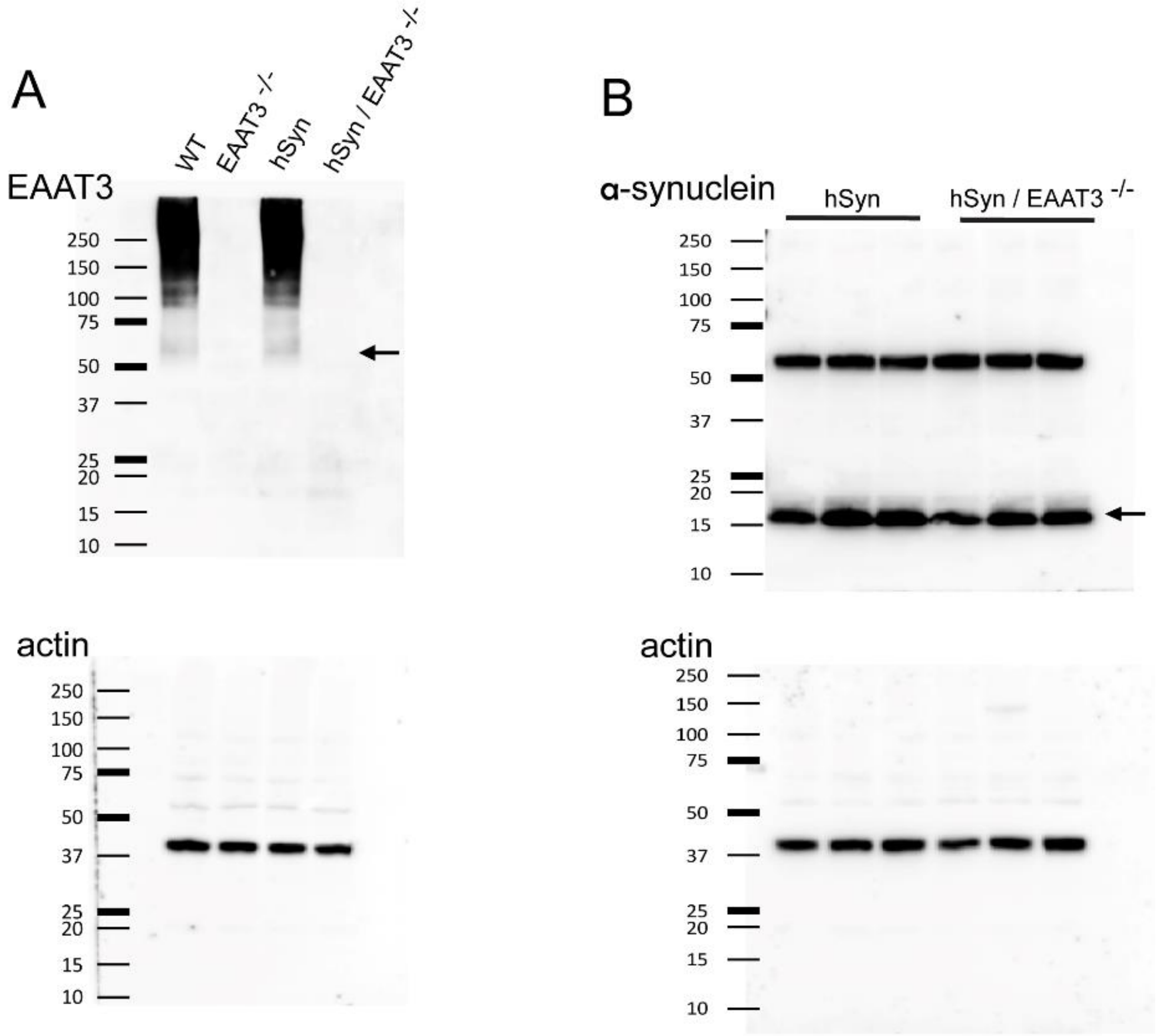


Supplemental Figure S1. Western blots of EAAT3 and human α-synuclein from brains of the transgenic mouse strains. (**A)** Immunoblots for EAAT3 in the four mouse strains. Arrow denotes the monomer band, with most EAAT3 protein present as higher molecular weight oligomers. The corresponding actin immunoblot was prepared from the same gel. Representative of two studies with similar results. (**B)** Immunoblot for human α-synuclein in three brains of the hSyn and hSyn / EAAT3$^{-/-}$ double-transgenic strain. The arrow denotes the monomer band. The corresponding actin immunoblot was prepared from the same gel.

**Supplemental Figure S2**

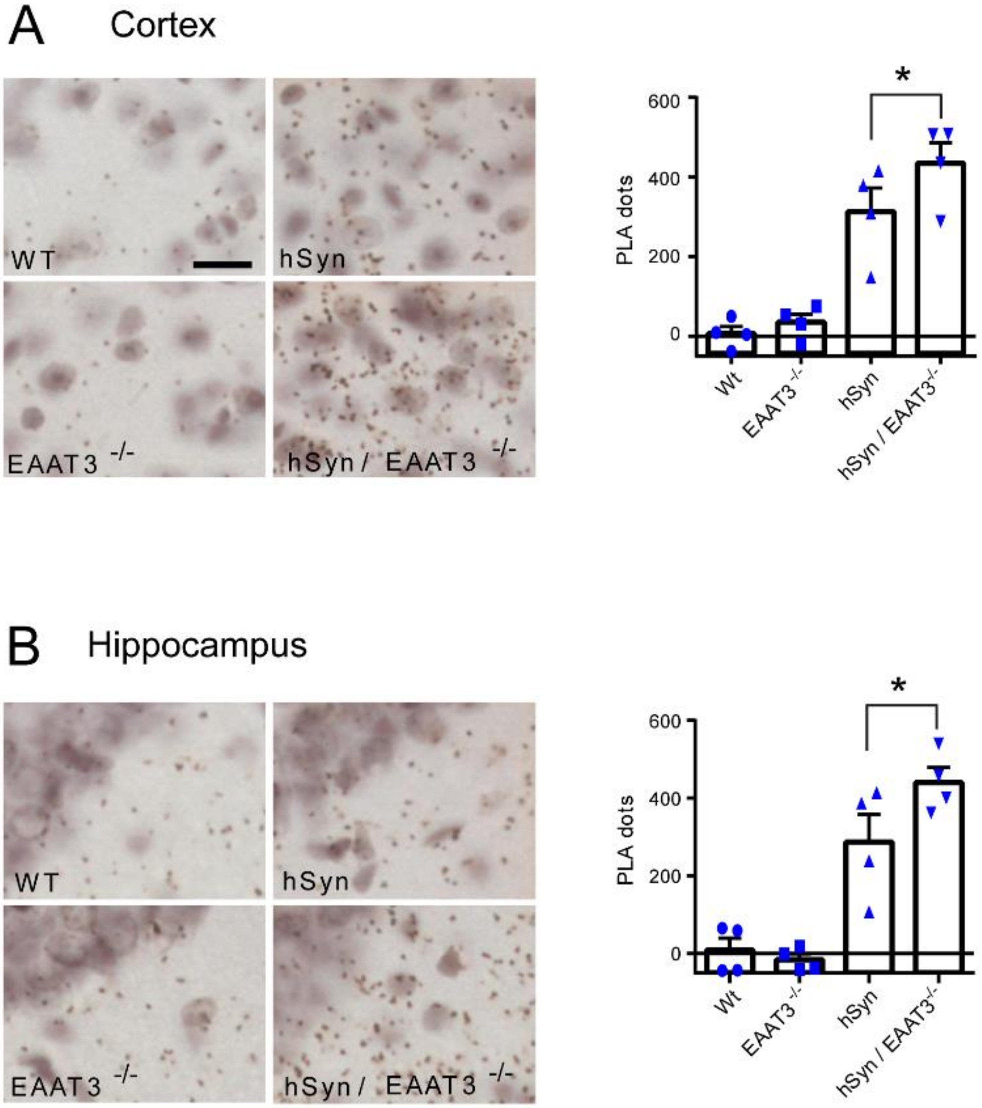


<u>Supplemental Figure 2.</u> Proximity ligation assay assessments of 4-hydroxynonenal-α-synuclein conjugates in neurons of (**A**) cortex and (**B**) hippocampus CA1 cell field. Co-localized 4-hydroxynonenal and α-synuclein are seen as small puncta interspersed around the larger cell nuclei. Scale bar = 20 µm. Graphs show relative number of puncta in the four moue strains. In each case the mean number of puncta counted in the Wt brains (dots / mm$^2$) were subtracted from all groups. n = 4; * $p < 0.05$.
